# A cilia-bound unconventional secretory pathway for *Drosophila* odorant receptors

**DOI:** 10.1186/s12915-024-01877-2

**Published:** 2024-04-12

**Authors:** Najat Dzaki, Mattias Alenius

**Affiliations:** Department of Molecular Biology, Umeå University, Umeå, 901 87 SE Sweden

**Keywords:** Olfactory sensory neurons, Odorant receptor, Soma to cilia trafficking, Unconventional secretion, Grasp65, *Drosophila*

## Abstract

**Background:**

Post-translational transport is a vital process which ensures that each protein reaches its site of function. Though most do so via an ordered ER-to-Golgi route, an increasing number of proteins are now shown to bypass this conventional secretory pathway.

**Results:**

In the *Drosophila* olfactory sensory neurons (OSNs), odorant receptors (ORs) are trafficked from the ER towards the cilia. Here, we show that Or22a, a receptor of various esters and alcoholic compounds, reaches the cilia partially through unconventional means. Or22a frequently present as puncta at the somatic cell body exit and within the dendrite prior to the cilia base. These rarely coincide with markers of either the intermediary ER-Golgi-intermediate-compartment (ERGIC) or Golgi structures. ERGIC and Golgi also displayed axonal localization biases, a further indication that at least some measure of OR transport may occur independently of their involvement. Additionally, neither the loss of several COPII genes involved in anterograde trafficking nor ERGIC itself affected puncta formation or Or22a transport to the cilium. Instead, we observed the consistent colocalization of Or22a puncta with Grasp65, the sole *Drosophila* homolog of mammalian GRASP55/Grh1, a marker of the unconventional pathway. The numbers of both Or22a and Grasp65-positive puncta were furthermore increased upon nutritional starvation, a condition known to enhance Golgi-bypassing secretory activity.

**Conclusions:**

Our results demonstrate an alternative route of Or22a transport, thus expanding the repertoire of unconventional secretion mechanisms in neurons.

**Supplementary Information:**

The online version contains supplementary material available at 10.1186/s12915-024-01877-2.

## Background

The cilium is a specialized organelle towards which receptor proteins are transported to facilitate and regulate signaling outputs. Although the intra-ciliary mechanisms of their movement are increasingly well-understood, how cilia-targeted proteins are trafficked from the soma to the cilium base remains understudied. In olfactory systems, the discrimination of odors is made possible by odorant receptors (ORs) localized to dendritic cilia. An OR is specific to its olfactory sensory neuron (OSN) and is among the highest expressed proteins in the cell. Both vertebrate and insect ORs are retained within the endoplasmic reticulum (ER) when expressed heterologously in cells lacking cilia [1–3]. This suggests that ciliated sensory neurons might have devoted special transport paths for receptors.

In the traditional view of transmembrane protein transport, nascent polypeptides must travel through several major cytoplasmic organelles prior to secretion. Briefly, newly synthesized proteins are packaged into COPII-encapsulated vesicles in the endoplasmic reticulum (ER), before emerging from ER exit sites to fuse with the cis-Golgi interface [4–6]. After passing through the intermediary Golgi stacks, they are packed into specialized secretory vesicles at the trans-Golgi for their journey towards the plasma membrane [7]. The process is heavily dependent on “tags” acquired throughout, including sugar and lipid moieties, which allow them to be sorted prior to further transport [8–10]. Ultimately, it is the gradual collection and exchange of such membrane tags found on these transport vesicles which will determine the direction and final destination of cargo proteins [11, 12].

However, the past two decades has seen the discovery of a growing number of unconventional protein secretion (UPS) pathways, many of which are indeed observed in neurons [13–15]. It is easy to see the logic behind the need for diversifying transport routes in neurons. Their unique morphology not only means long distances between the nucleus and sites of protein function along the dendrite but also the need for organized bipolarity in transport direction. In hippocampal neurons, the ER is shown to be contiguous throughout the axon and dendrite. Here, the mRNAs of a number of secreted factors and membrane proteins are preserved and translated pro rata where necessitated by neuronal development and maintenance [16, 17]. Curiously, the Golgi apparatus is largely absent from dendrites [18, 19], and the neuronal membrane was shown to be enriched with proteins bearing ER-associated glycosylation characteristics [20], suggesting that considerable levels of UPS indeed take place in neurons.

In non-neurons, UPS is typically initiated under duress. In yeast, UPS occurs within compartments of unconventional secretion (CUPS), the formation of which takes place through various ways. Whereas protein misfolding induces UPS in the ER, during starvation, the formation of CUPS is driven by proteins of the macroautophagy (hereby autophagy) process [21, 22]. The transport of the much investigated UPS cargo protein IL-1β involves components from various canonical cellular pathways such as exosomes [23], multi-vesicular bodies (MVBs) [24], and secretory (Sec) bodies [25]. Interestingly, even if induced by different agents of different transport systems, some common criteria define UPS, such as the presence of *Golgi reassembly and stacking protein* (GRASP) protein orthologs [26–30], as well as the glycosylation patterns of their cargo proteins.

Here, we present evidence that OR transport to the cilium is routed through a UPS pathway in *Drosophila melanogaster*. We show that under standard growth conditions, Or22a consistently amasses into punctate structures at two points within the OSN. A large, single punctate is observed at the soma body exit, whereas multiples of smaller Or22a puncta form in the dendrite prior to its cilia base. With *green fluorescent protein* (GFP)-tagged markers of the ER-Golgi apparatus, we show that neither of the Or22a puncta colocalize significantly with Golgi and that loss of proteins integral to the conventional anterograde process neither abrogated puncta nor negatively impacted Or22a transport to the cilium in Or22a OSNs. Intriguingly, the proteins regulating cilia transport do not colocalize with Or22a puncta, suggesting that the cargo and the transporter of the cilia compartment are transported differently. Or22a puncta are then shown to be associated with Grasp65, the sole GRASP ortholog in *Drosophila*. Finally, we show that this unconventional pathway of Or22a transport is induced during starvation, suggesting that the early routing from the ER of Or22a into its UPS pathway abets a state-dependent response via transport regulation.

## Results

### Or22a does not relate to conventional transport organelles

**Fig. 1:**
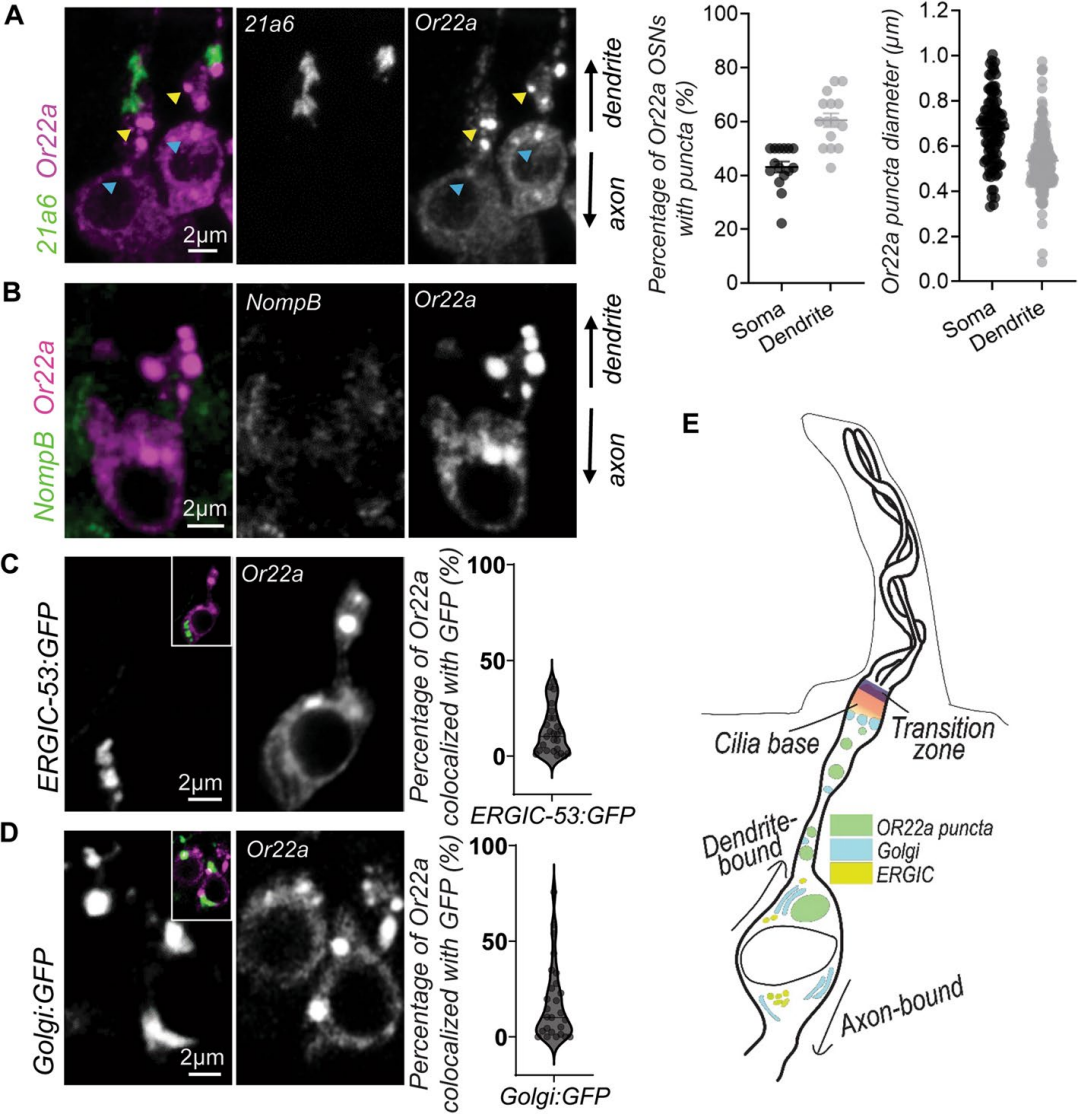
Or22a puncta are observed at stereotypical sites and do not significantly overlap with ERGIC-53 or Golgi. **A** Or22a puncta typically manifest as a singular, large structure at the cell body exit of the soma (blue arrowheads) or multiples of two to four (yellow arrowheads) prior to the cilia base, here marked by 21a6. Accompanying graphs show the percentage of Or22a OSNs with puncta (for each, *n* = 15 antennas) or the average puncta sizes in either the soma (*n* = 102 puncta) or dendrite (*n* = 221 puncta). **B** Or22a puncta do not co-localize with NompB, the *Drosophila* homolog of the cilia transport protein IFT88. **C**, **D** Or22a puncta also show no significant association to proteins of conventional transport organelles, i.e., ERGIC-53 (**C**, *n* = 31) or Golgi (**D**, *n* = 33) structures, where *n* represents the total number of puncta analyzed. Inset are dual-channel counterparts of split images. The GFP-tagged proteins are in green; Or22a is in magenta. **E** A simplified representation of Or22a puncta transport within its OSN. Not drawn to scale

In *Drosophila*, ORs are transported with an established directionality heavily favoring the cell’s dendritic end, where their receptor function takes place. Immunohistochemistry indicated that Or22a follows a stereotypical path towards the cilia compartment. Approximately half of Or22a-expressing OSNs (43.11% ± 7.86%, *n* = 15) contained one large Or22a punctate at the cell body exit into the dendrite (0.6775 ± 0.1548 μm in diameter). These neurons would always display a second set of two to four puncta near the dendritic cilia base (0.5348 ± 0.1220 μm; Fig. 1A). However, more OSNs would bear dendritic puncta (60.48% ± 9.77%) than they would somatic puncta. Regardless, several GFP-tagged marker proteins of cilia transport proteins (*intraflagellar transport* (IFT) and *Bardet-Biedl syndrome* (BBS)) and cilium assembly complexes were relatively poorly colocalized to Or22a puncta, indicating that the OR and the traditional cilia transport machineries take different routes to the cilium (Fig. 1B and Additional File 1: Figure S1).

To pinpoint the origin of the Or22a puncta, we examined Or22a colocalization with various GFP-tagged markers of conventional transport organelles. In conventional transport, proteins sequentially proceed from the ER to Golgi, often through the ER-Golgi intermediary compartment (ERGIC). Or22a puncta overlapped only modestly with the ERGIC marker ERGIC-53 (13.06 ± 12.14%; Fig. 1C) and Golgi-stack proteins (16.73 ± 18.83%; Fig. 1D), results that indicate an incomplete reliance of Or22a on conventional transport. Comparative distribution analysis further revealed that both ERGIC-53 and Golgi markers were more heavily localized in the axonal half of the OSN cell body (Additional File 1: Figures S2A and S2B). Apart from suggesting that a strong polarization in transport exists in the OSNs, these initial observations together indicate that Or22a undertake an alternative pathway towards the cilia, one which occurs in an ERGIC-53 and Golgi-independent manner (Fig. 1E).

### COPII-vesicle proteins are dispensable for the formation of Or22a puncta and its transport to the cilia

**Fig. 2:**
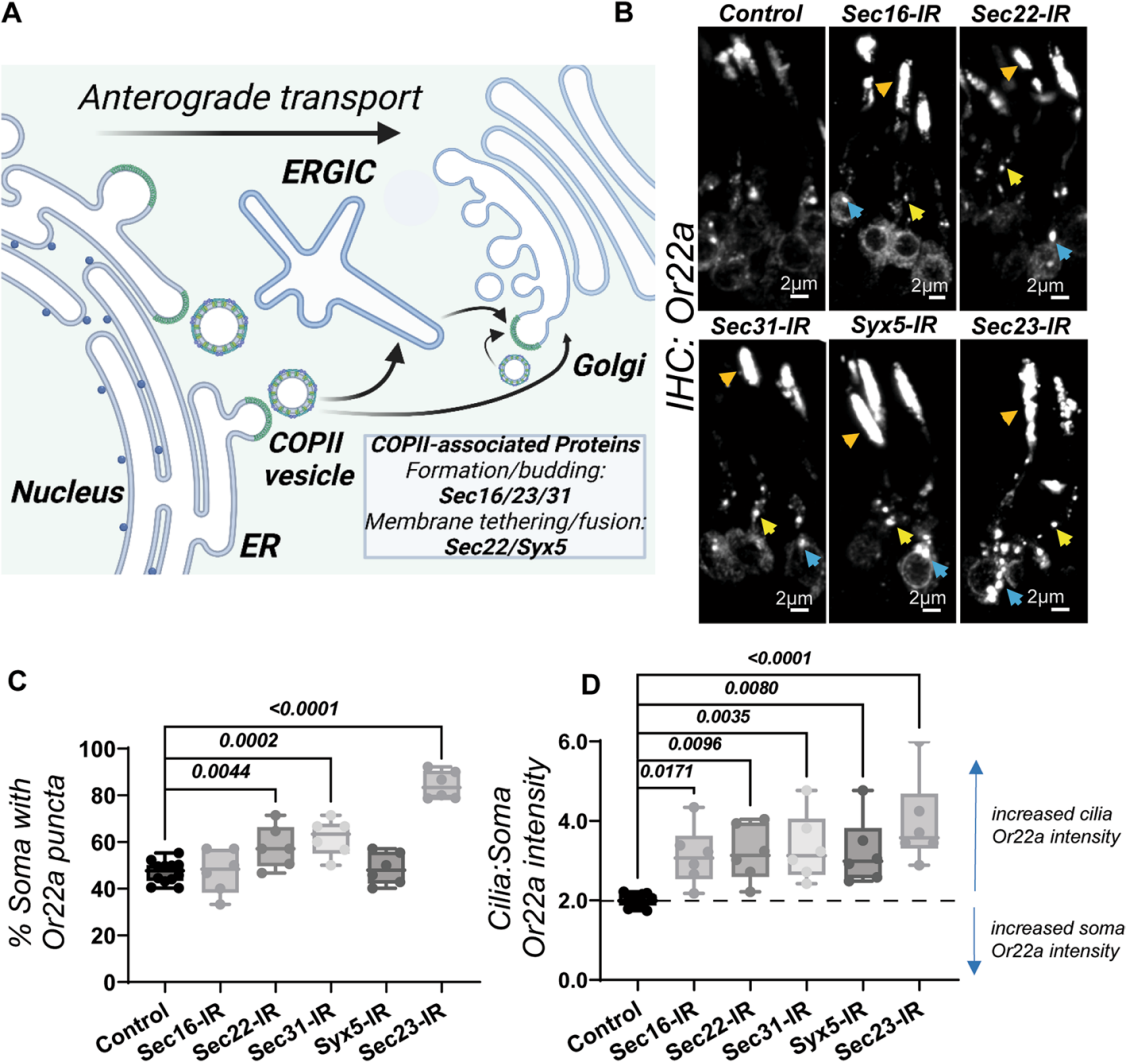
Or22a soma to cilia transport is enhanced where conventional ER to Golgi trafficking is disrupted. **A** A simplified diagram of the conventional protein trafficking process. Proteins of the COPII anterograde transport machinery are listed. Figure produced on BioRender.com. **B** The knockdown of COPII genes in OSNs did not abrogate Or22a puncta at either soma (blue arrows) or dendrite (yellow arrows). For many, cilia Or22a levels were instead intensified (orange arrows), an indication of heightened cilia-bound traffic. **C** Quantitative analyses for Or22a puncta frequency across a population of Or22a OSNs. **D** Quantitative analyses for Or22a intensity ratios in cilia vs soma. For control, *n* = 12; for other genotypes, *n* = 6, where *n* is the total number of antennas analyzed; error bars represent mean SEM. One-way ANOVA with multiple comparison test and Dunnett’s correction was applied for statistical analysis

Proteins of the *coat protein complex* (COP) group form the vesicles which transport cargo from the ER to ERGIC or Golgi, and vice versa. COPII proteins such as Sec22, Sec23, and Sec31 are specifically involved in anterograde transport (Fig. 2A). Should Or22a puncta have any formative connections to COPII, we reasoned that eliminating COPII genes from OSNs would negatively affect both puncta numbers and overall Or22a transport into the cilia. Interestingly, singular knockdown of COPII proteins failed to abrogate either the soma or dendrite Or22a puncta (Fig. 2B). Instead, OSNs lacking Sec22, Sec23, or Sec31 displayed more Or22a-positive puncta (Fig. 2C). Further analysis showed that the knockdown of these COPII genes also resulted in significantly higher ciliary Or22a levels (Fig. 2D), indicating that Or22a transport to the cilium was not negatively affected but rather increased.

This inverse relationship between soma to cilia intensity ratios was especially prominent with Sec23 knockdown (Fig. 2B, D). Along with its binding partner Sec24, Sec23 forms the inner core layer of COPII vesicles and is indispensable to anterograde transport [31]. Sec23 loss increased both Or22a somatic puncta frequency and its cilia intensity (Fig. 2C, D), outcomes which strongly support the hypothesis that conventional, COPII-mediated transport is dispensable for the soma-to-cilia trafficking of Or22a.

### OR and Orco take separate transport routes in the soma

The functionality of a *Drosophila* OR lies in its heteromerization with the common OR co-receptor Orco [32]. It is long assumed that ORs in fact require Orco to be successfully transported and that the coupling of the two proteins takes place in the soma, an association maintained into the cilia. In control antennas, Orco often colocalizes with Or22a puncta. However, our initial analysis revealed instances in the OSN cell body where they appeared to be separate entities (Additional File 1: Figure S3A). We had previously determined the colocalization percentage between Or22a and Orco to be relatively high (64.37 ± 14.13%; Additional File 1: Figure S1A), although this was a lower value than expected. Histogram profiles from sections across different Or22a puncta then demonstrated the varied degrees of overlapping occurring between Or22a and Orco (Figure S3B). A more critical analysis separating the somatic and dendritic puncta cohorts revealed that the Or22a and Orco colocalize significantly more closely in the dendrite (70.02 ± 20.79%; *n* = 129) than they do in the soma (51.47 ± 26.53%; *n* = 126; Additional File 1: Figure S3B).

**Fig. 3:**
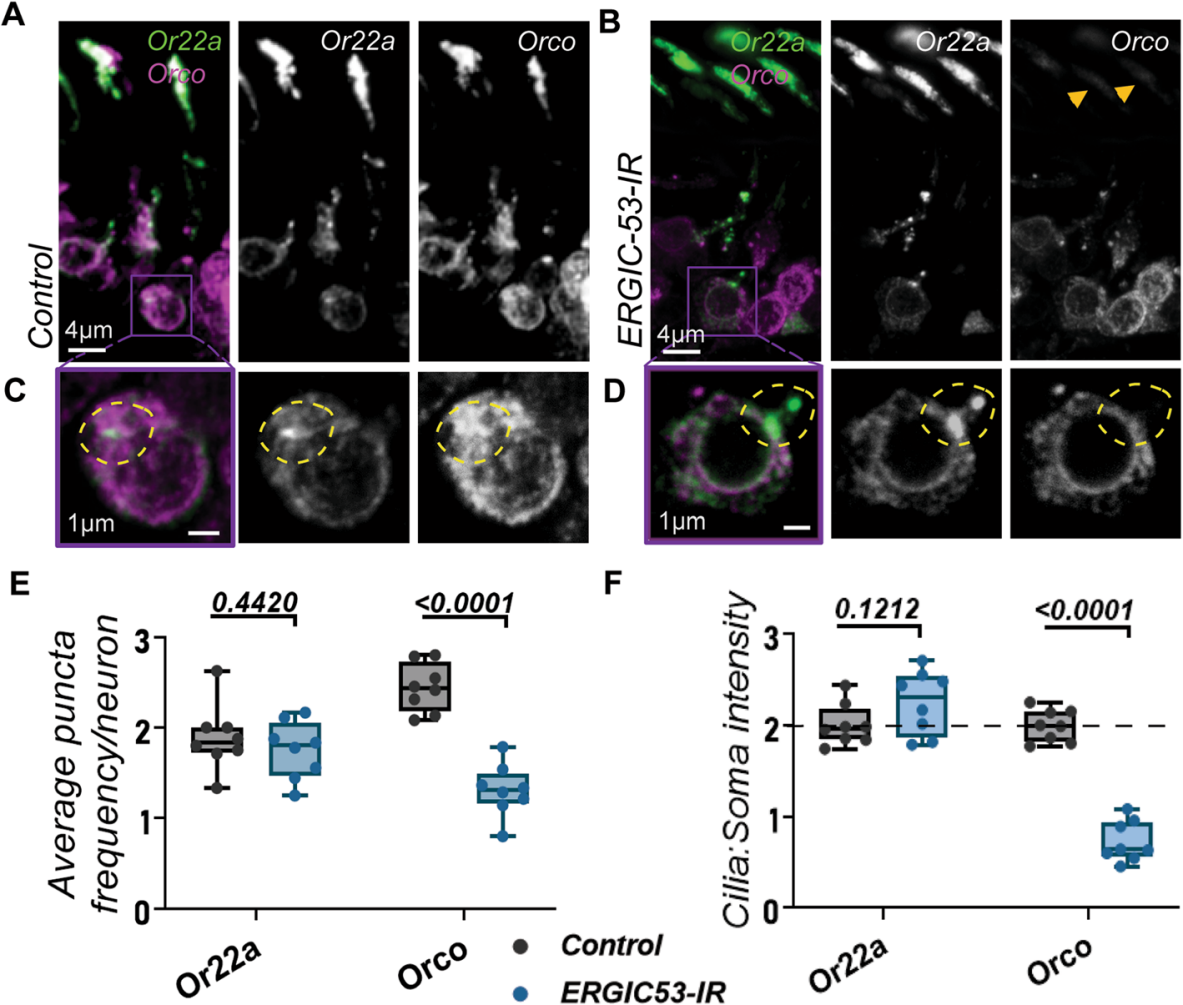
Loss of conventional ER to Golgi transport via ERGIC-53 disruption does not negatively affect Or22a packing into puncta nor its overall transport. **A**, **B** *ERGIC-53*-knockdown almost completely diminishes Orco in the cilia (orange arrowheads), but Or22a is not significantly affected. **C**, **D** Magnification of isolated regions (purple squares) demonstrates that the Or22a puncta-mediated transport continues in spite of Orco puncta abrogation (circled yellow). **E** Orco puncta frequency is significantly reduced in *ERGIC-53* knockdown antennas in comparison to controls, but Or22a remains unchanged. **F** Orco, but not Or22a, fails to be transported into the cilia and is retained in the soma in *ERGIC-53*-IR neurons. Control *n* = 8; *ERGIC-53*-IR *n* = 8. For all, error bars represent mean SEM. Two-tailed Student’s *t*-test was applied for statistical analyses

We therefore questioned if the initial Orco and Or22a transport could be routed into different paths. Consistent with this hypothesis, ERGIC-53 knockdown from the OSN affected Orco negatively but not Or22a (Fig. 3A, B). Furthermore, the disappearance of Orco puncta in knockdown flies did not abrogate Or22a puncta (Fig. 3C, D), and a reduction of Orco puncta frequency but not that of Or22a was duly observed (Fig. 3E). An overall transport block of Orco in Or22a OSNs due to the loss of ERGIC-53 was made clearer by the near absence of Orco in the cilia, even though Or22a intensity levels there remained relatively unchanged (Fig. 3F). These results together not only strengthen the case for an unconventional element in Or22a trafficking, but challenges the widely accepted notion that insect odorant receptors are entirely dependent on Orco for their transport.

### Or22a take a Grasp65 mediated unconventional protein transport pathway

**Fig. 4:**
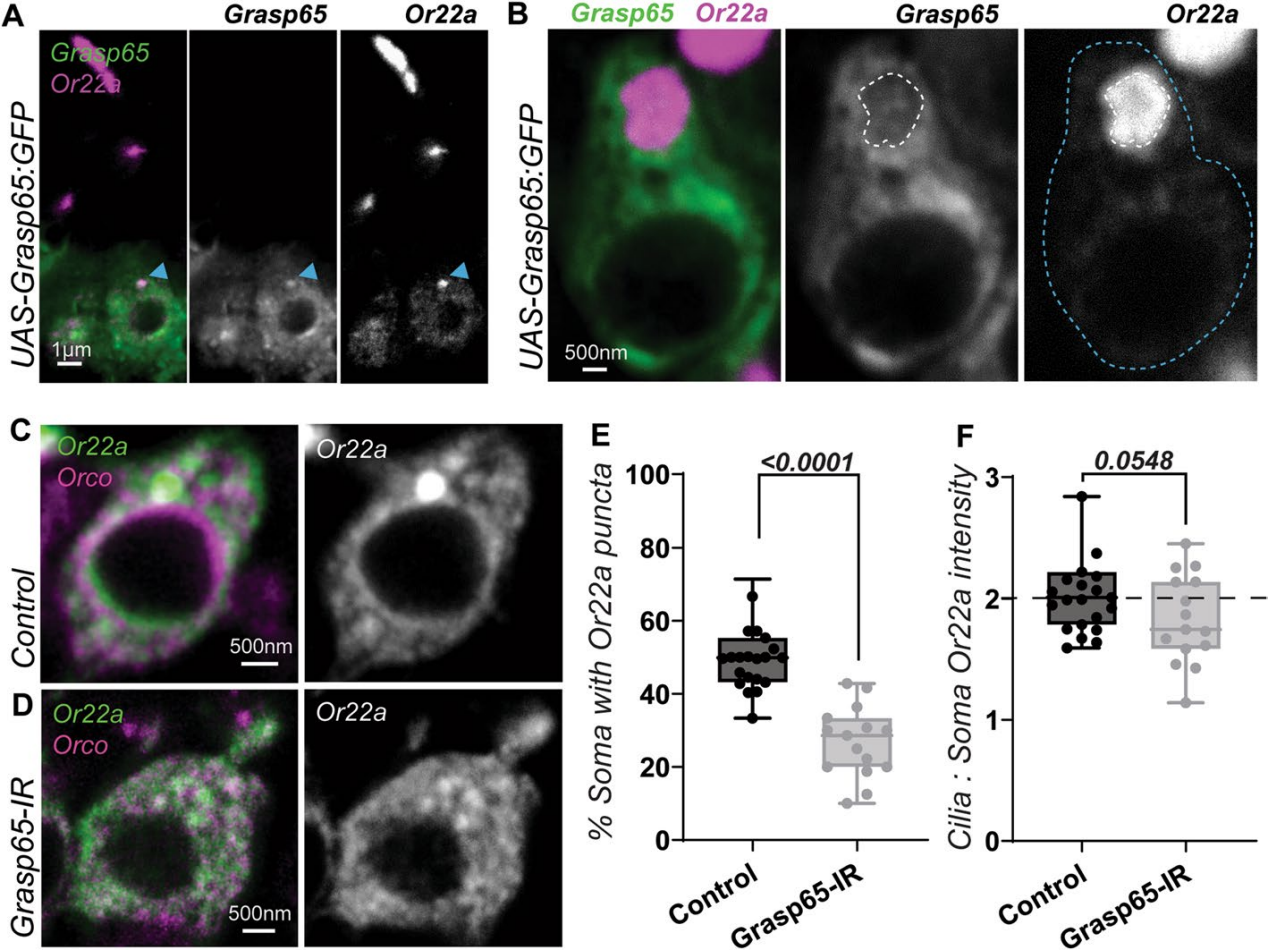
Or22a is transportable through a Grasp65-dependent unconventional route of protein secretion. **A** The *Golgi reassembly and stacking protein 65* (Grasp65) is a marker of unconventional secretion. Somatic Or22a puncta shown here is colocalized with Grasp65 (blue arrowheads). **B** Magnification of an Or22a OSN bearing enlarged Or22a puncta. White dashed lines indicate an Or22a punctate. Blue dashed lines indicate the somatic body. Grasp65 as well as Or22a concentrate with a bias around the presumed punctate membrane. **C**, **D** Grasp65 knockdown largely abrogates soma Or22a puncta. Magnification of an Or22a OSN in a *Grasp65*-IR antenna reveals a void in the space where the soma puncta typically manifests. **E**, **F** Quantitative analyses for Or22a puncta formation and comparative cilia to soma Or22a intensity in *Grasp65-*IR flies. For controls, *n* = 23; for *Grasp65*-IR, *n* = 15. For all, error bars represent mean SEM. Two-tailed Student’s *t*-test was applied for statistical analyses

The incomplete reliance of Or22a on Golgi transport shown thus far implicates a secondary transport pathway for the ORs. In recent years, Grasp65 has evolved to become a marker of UPS transport vesicles. Despite its name and the involvement of its mammalian orthologs in Golgi cisternae organization, this aspect of its function is poorly conserved across kingdoms, and Golgi-stacking may not be the primary purpose of Grasp65 [33]. Moreover, its depletion in other eukaryotes neither affects Golgi function nor structural integrity [34, 35]. The loss of GrpA, the Grasp65 ortholog in the slime mold *Dictyostelium discoideum*, instead leads to cytoplasmic accumulation of UPS secreted proteins, despite an intact Golgi [33]. This discovery was significant as it conveys that a Grasp65-like protein is crucial to the UPS process. Through the overexpression of a GFP-tagged version of Grasp65, we subsequently observed its extensive colocalization with somatic Or22a puncta (Fig. 4A). Upon further inspection, Grasp65 was seen surrounding the Or22a-heavy punctate, suggesting that Grasp65 may be membrane-bound, as befitting its tethering function (Fig. 4B).

Grasp65 knockdown with *Peb-*Gal4, a driver activated in all OSNs, caused a frequency loss of somatic Or22a puncta (Fig. 4C, D, E and Additional File 1: Figure S4A), further supporting the notion that Grasp65 proteins are involved in at least the formation of soma-body exit puncta. Although the cilia to soma Or22a intensity of *Grasp65-*IR flies was not altered in comparison to controls (Fig. 4F and Additional File 1: Figure S4A), a downward trend was apparent. We thus proceeded to knock *Grasp65* down specifically within Or22a OSNs with an *Or22a*-Gal4 driver (Additional File 1: Figure S4B). The loss in somatic Or22a puncta frequency was replicated, affirming the critical role of Grasp65 in their formation. In these *Or22a*-Gal4 > *Grasp65-*IR flies, the magnitude of reduction in cilia to soma Or22a intensity was also significantly observed. These outcomes together show that Or22a carried within such vesicle-like somatic puncta is transported towards and terminalizes within the cilia. More crucially, it prescribes a functionality to this Grasp65-mediated pathway as an unconventional secretory route in the overall regulation of *Drosophila* OR transport.

### Or22a is digestible by Endo H

Most proteins freshly exiting the ER carry a high-mannose, *N*-linked oligosaccharide core. Within the Golgi, this is trimmed off and replaced with more sugar units, resulting in a protein with a complex *N*-glycan terminus [36]. Importantly, proteins which do not traverse through the Golgi would not acquire a key modification that confers a resistance to endoglycosidase H (Endo H) [37]. The sensitivity of a protein towards this enzyme has thus been applied to assess Golgi bypass [38, 39]. To further verify if Or22a is indeed susceptible to unconventional secretion, we subjected antennal lysates from *w*^*1118*^ flies to Endo H digestion prior to blotting. Two distinct bands were highlighted by the purified anti-Or22a antibody (Additional File 1: Figure S5A). A larger ~ 58 kDa band was probed, along with a ~ 42 kDa band most likely representing the monomeric Or22a protein (Additional File 1: Figure S5A). Both bands were significantly intensified upon Endo H digestion (Additional File 1: Figures S5A and S5B; raw images in Figure S7). This indicates that an Or22a species exceeding the 100 kDa maximum protein size resolved on the membrane can be processed into simpler forms. Importantly, it shows that a significant amount of the protein is maintained in a glycosylated state. Taken together, our immunohistochemistry, genetic, and biochemical results support the existence of a Golgi-bypassing pathway in Or22a transport.

### The unconventional transport pathway of Or22a is encouraged by starvation but not a high-sugar diet

**Fig. 5:**
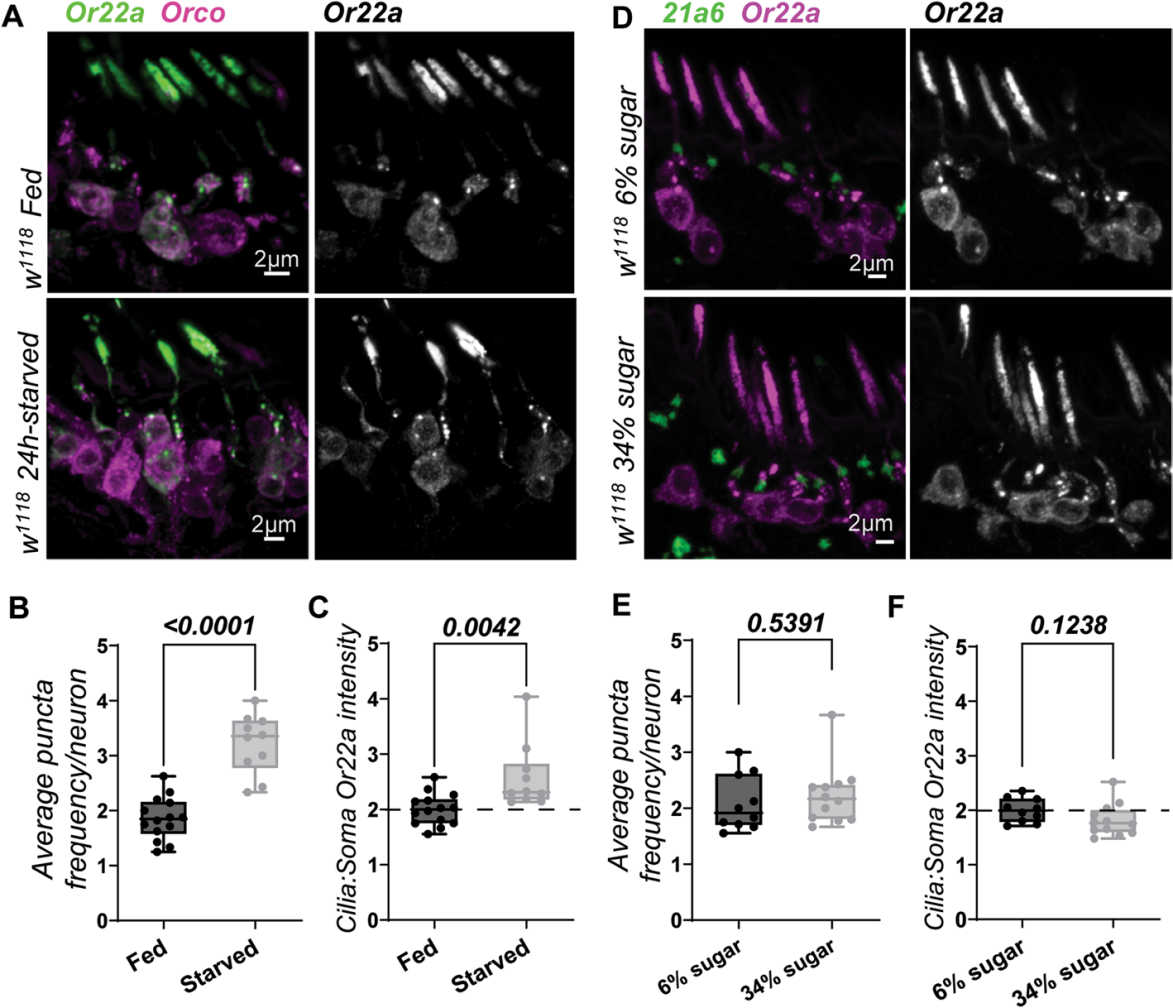
Starvation, not sugar overconsumption, encourages the unconventional transport of Or22a. **A** Or22a staining in *w*^*1118*^ flies in comparison to those deprived of food for 24 h. **B** A general increase in Or22a puncta frequencies were observed in starved flies. **C** Soma Or22a intensity levels are lower in starved than in fed flies, an implication that Or22a puncta are functional vesicles for its transport into the cilia. For both **B** and **C**, fed *n* = 14; starved *n* = 10. A high-sugar diet, known to alter peripheral neuron activity, had no immediate effects on Or22a transport. **E**, **F** Quantitative analyses of puncta frequency and cilia to soma intensity in flies fed a control or high-sugar diet. For 6% sugar, *n* = 10; for 34% sugar, *n* = 12. For all, error bars represent mean SEM. Two-tailed Student’s *t*-test was applied for statistical analyses

Approximately half of the OSNs that express Or22a carried the somatic Or22a puncta (Fig. 1A), indicating that not all cells at a given time point use the UPS pathway under normal conditions. Most identified cases of UPS take place under cellular duress. We therefore addressed if the Or22a puncta is increased by stress and from food deprivation in particular [40]. We found that 24-h wet starvation indeed induced a concurrent increase in Or22a puncta numbers (Fig. 5A, B) and the cilia intensity of the receptor (Fig. 5C) in control flies, suggesting greater Or22a transport.

Other nutritional states also regulate olfaction. We recently showed that a high-sugar diet suppresses the odor response of Or22a OSNs [41]. However, no discernible differences were observed between flies on control food with a 6% sugar content and those on the same food supplemented with sucrose to a 34% sugar content level (Fig. 5D, E), even after a prolonged 4-day period of feeding. Puncta formation is thus independent of dietary sugar levels, and sugar-induced modulation might instead occur at the cilia entry step or through OR function in the cilium. Our results thereby suggest that nutritional scarcity, rather than a high-sugar diet, enhances the transport of Or22a via an unconventional pathway.

### Grasp65, but not GM130 or intact somatic Golgi, is required for Or22a puncta formation

When Grasp65:GFP overexpressing flies were then starved, we not only observed the expected increase in puncta frequency but that these were both Or22a and Grasp65 positive (Additional File 1: Figures S6A and S6B), supporting the argument that Or22a puncta are enlarged unconventional transport structures. We also reasoned that should they be Golgi-arising, then starvation would significantly increase the overlap between Or22a puncta and Golgi stacks. Instead, colocalization analysis with the *cis*-Golgi marker GM130 showed no discernible differences between control and starved flies (Additional File 1: Figures S6C and S6D), further dissociating the involvement of the Golgi apparatus from Or22a puncta formation.

**Fig. 6:**
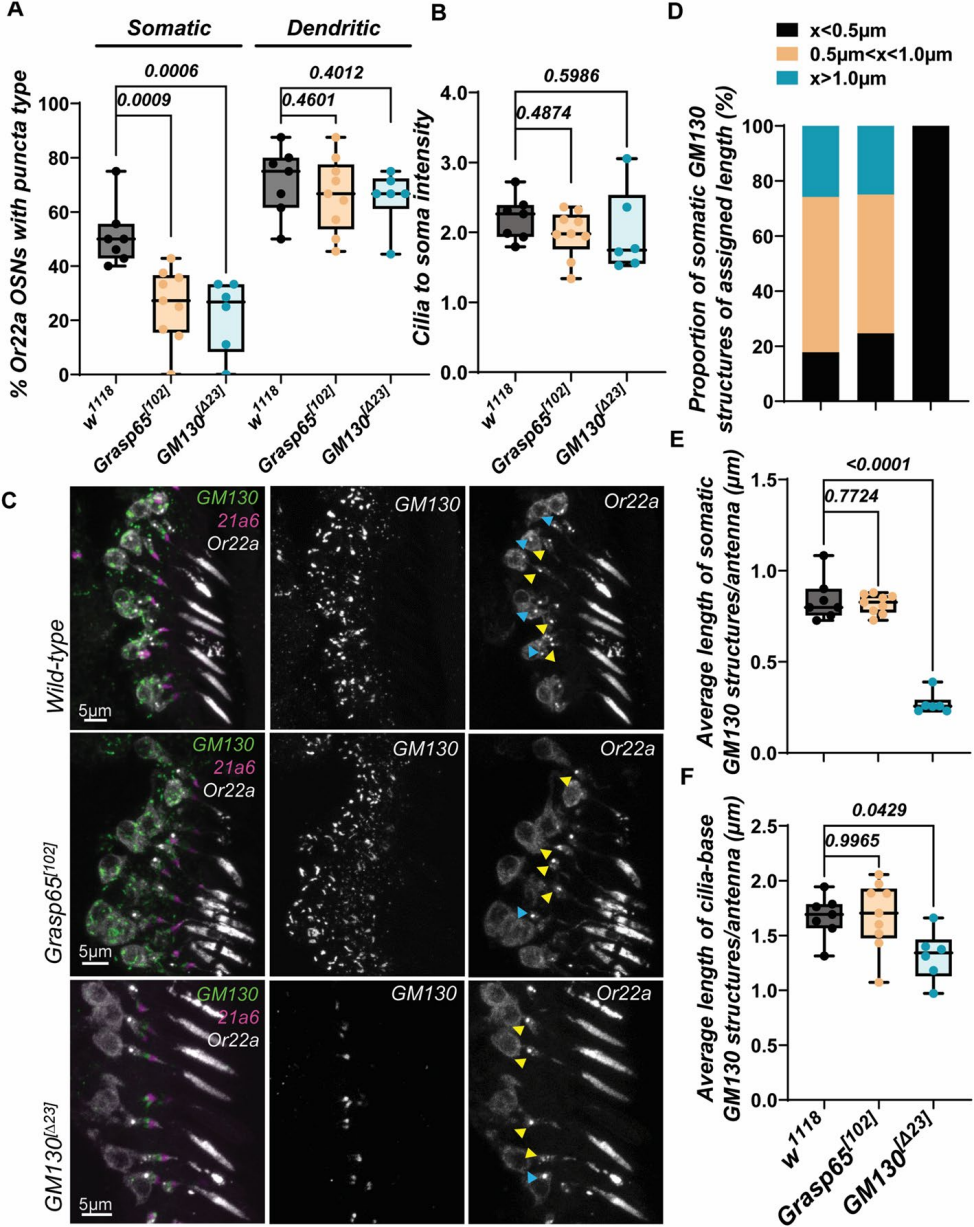
Grasp65, but not GM130 or intact somatic Golgi, is required for Or22a puncta formation. **A**, **B** Somatic Or22a puncta are lost in *Grasp65*^*[102]*^ and *GM130*^*[∆23]*^ mutants, with no changes to dendritic puncta or cilia vs soma Or22a localization. For Wild-type, antennal *n* = 7; for *Grasp65*^*[102]*^, *n* = 9; for *GM130*^*[∆23]*^, *n* = 6. **C** Representative images of GM130 distribution in control *w*^*1118*^ or homozygous *Grasp65*^*[102]*^ and *GM130*^*[∆23]*^ mutants. GM130 is a *cis-*Golgi localized membrane protein without which the Golgi could not compartmentalize into *cis*, *medial*, and *trans* stacks and is thus often used as a marker to convey Golgi apparatus integrity. Here, although somatic Golgi stacks are completely ablated *GM130*^*[∆23]*^ mutants, they are retained in *Grasp65*^*[102]*^ mutants, despite the reputed function of Grasp65 as a Golgi stacking protein. **D**–**F** The integrity of somatic and dendritic GM130-positive structures is compromised in *GM130*^*[∆23]*^ but not *Grasp65*^*[102]*^ mutants. In **D**, Wild-type somatic structures measured were *n* = 225; for *Grasp65*^*[102]*^, *n* = 377; for *GM130*^*[∆23]*^, *n* = 118. In **E** and **F**, Wild-type, antennal *n* = 7; for *Grasp65*^*[102]*^, *n* = 9; for *GM130*^*[∆23]*^, *n* = 6. Soma puncta are marked by blue arrowheads, and dendritic puncta by yellow arrowheads. Scale bars are as shown. Error bars represent mean SEM. One-way ANOVA with multiple comparison test and Dunnett’s correction was applied for statistical analysis

Such phenotypes led to the question of whether Or22a puncta are lost in *Grasp65* mutants and, should it be so, if this is related to the integrity of the Golgi body. Indeed, *Grasp65* mutants showed a dramatically reduced frequency of soma Or22a puncta, with a downward if minor reduction in ciliary Or22a intensity (Fig. 6A, B). Remarkably, this occurred without compromising the structural integrity of Golgi stacks, as revealed by both immunohistochemistry and quantitative GM130-positive structures analyses (Fig. 6C–F). This is in complete contrast to *GM130* mutants which, despite also bearing significantly fewer somatic Or22a puncta, displayed a near complete ablation of their antennal Golgi bodies. Together, these observations clearly demonstrate that somatic Or22a puncta is an inherently Grasp65-dependent structure and that the loss of Grasp65 from intact Golgi stacks is sufficient in preventing puncta formation from taking place.

## Discussion

While pre-cilia entry gatekeeping is well-studied, it is less clear how cilia cargos are sorted and reaches the pre-cilia region. Here, we show that OR transport from the soma to the cilium is strictly regulated and stereotypical between cells, with Or22a-positive puncta exhibiting two distinct “checkpoints” in its OSN. The first is found at the soma cell body to dendrite exit, while the second is observed short of the cilia base in the proximal dendrite. Interestingly, this route is not taken by other cilia proteins, as we demonstrated that the main components of intraflagellar transport (IFT88/NompB), cargo retrieval (BBS1 and BBS8), and cilia base assembly (Cc2d2a) systems do not colocalize with Or22a. Higher Or22a puncta frequency was also found to translate into higher cilia Or22a intensity. These observations complement our hypotheses that such puncta serve as transport and storage vehicles for certain pre-sorted cilia cargo including ORs and that they help regulate the influx of cilia membrane proteins in and out of the cilium.

Most unconventional transport pathways are connected to stress [33, 42]. In mammalian systems, increasing evidence suggest a central role of GRASP55 in mediating cellular conditions to the need for UPS [43, 44]. In the adult *Drosophila*, starvation is known to drive hyperactivity and foraging behavior via octopamine [45]. Somatic Or22a puncta extensively overlapped with Grasp65, and we observed higher somatic Or22a puncta and cilia levels in starved flies, an indication that nutrient scarcity could induce UPS and thereby increase the ciliary accumulation of Or22a. Together, these findings propose the attractive notion that under pressing conditions, Grasp65-mediated UPS would mediate the influx of ORs into the cilia and thus propel food-foraging tendencies, likely by increasing olfactory sensitivity. This operates under the presumption that there is a positive relationship between increased ciliary Or22a to electrophysiological and behavioral responsiveness, both of which are already shown to be abolished in singular OR mutants [46–48]. Unexpectedly, a recent report shows that rather than invoking a sustained electrical potential, odors at very high concentrations completely silences their corresponding OSN [49]. Electrophysiology and behavior data on the changes which occur under OR overexpression or flooding are therefore required to expose a tangible link between Grasp65-mediated UPS to phenotypes that would most benefit the fly.

Or22a shares its dendritic UPS route with its co-receptor Orco. However, immunohistochemistry and colocalization analyses suggest that Or22a and Orco may be discretely independent entities from one another in the soma. We show that Orco leaves the ER, colocalizes with ERGIC, and requires ERGIC-53 to associate with Or22a at the soma exit. The initial difference in transport is consistent with the argument that Or22a and Orco transport is differentially regulated [50]. Orco also requires ERGIC-53 and its fusion with the soma Or22a puncta to get to the cilium. However, Or22a transport to the cilium is unperturbed in ERGIC-53 knock down flies. This was unexpected as Orco mutants of various insect species including *Drosophila*, mosquitoes, and ants [46, 51, 52] lose antennal OR proteins, leading to olfaction deficiencies. Orco mutants in various species have reduced OR expression, suggesting that Orco expression, translation, or soma localization is required to stabilize OR expression. ERGIC-53 in other species is shown to shuttle secreted proteins destined for unconventional transport [53]. Thus, our demonstration that Orco is ERGIC-53 dependent shows that the ERGIC organelle also routes transmembrane proteins into a UPS pathway.

The bulk transport of receptors to synapses or cilia is a grand task for neurons, especially given that ORs are among the highest expressed genes in OSNs [2]. A dedicated UPS route for ORs would therefore not only separate them from other dendrite proteins but would relieve the transport constrain off conventional paths. It also allows the neuron to regulate local OR levels without affecting other transport pathways, as we observed in starved flies. Importantly, as well as showing that cilia cargo proteins can be pre-sorted before reaching the periciliary region to facilitate onward transport, the OR-UPS route demonstrates that cilia transport regulation might have already begun in the ER.

It is notoriously difficult to transport ORs to the plasma membrane of cultured non-neuronal cells [1], possibly due to a lack of cilia on such cell lines. We have presented evidence of the connection between Or22a puncta and its transport into the cilia and that Or22a can be digested by endo H. Taking into account the consistent association between Or22a puncta and Grasp65, we propose that glycosylated Or22a is indeed carried in such UPS vesicles towards the cilia. Although the transport route may dictate OR glycosylation and transportability to the plasma membrane, this appears to be inherently determined by the presence of a cilia structure. Accordingly, the glycosylation state of vertebrate polycystin-1 and polycystin-2 have already been shown to direct the transport routes of these proteins to the ciliary or cellular plasma membrane [54, 55]. In *Drosophila*, Ir8a, the coreceptor for a second type of receptors, i.e., the ionotropic receptors, require N-glycosylation for proper cilia localization [56]. How exactly glycosylation affects the cilia transport of ORs remains a subject for investigation.

It also remains to be shown if the unconventional transport mechanism is conserved for ORs in vertebrate OSNs, particularly as significant structural differences exist between vertebrate and insect ORs [32]. Despite their dissimilarities, however, all ORs are transported to the cilium in a cilia-transport protein dependent manner [50, 57]. Several mice odorant receptors were moreover shown to undergo a unique sorting and transport process via dendritic MVB-like structures [58], albeit independently of mammalian GRASPs. Nonetheless, rat hippocampal neurons are already known to lack dendritic Golgi structures, to bear membranes enriched with ER-glycosylated proteins, and to utilize UPS to transport glutamate receptors and ion channels to synapses [59]. These observations together suggest that the UPS of receptors is indeed commonplace in different neurons from flies to vertebrates, highlighting the need for further research to broaden our understanding of their mechanisms.

## Conclusions

Here, we described a novel puncta-mediated transport route for Or22a in *Drosophila* OSNs. It fulfills the criteria of a bona fide UPS pathway as Or22a puncta do not relate to somatic or dendritic ERGIC and Golgi but are instead associated with the UPS marker Grasp65. Soma puncta and the cilia transport of Or22a are also unperturbed by the loss of several COPII proteins required for anterograde Golgi-dependent transport. Rather, the opposite is observed, as abrogation of COPII proteins increased Or22a transport and puncta formation. While Or22a and its co-receptor Orco eventually converge into this UPS route in the dendrite, we present evidence that their sorting into the pathway within the soma occurs via distinct means. We then show Or22a and Grasp65-positive puncta frequencies are enhanced due to nutritional deprivation, a condition known to encourage UPS, corroborating the argument that the OR route is a categorical UPS pathway. Altogether, these findings indicate that the OR-UPS route facilitates basal OR transport in parallel to the conventional Golgi-dependent route, thus highlighting the versatility in protein transport found within the olfaction system and neurons in general.

## Methods

### Fly stocks and sampling

Lines were obtained from either Bloomington (BDSC) or Vienna (VDRC) *Drosophila* stock centers or were gifts from collaborating labs. A complete list of genotypes and stock numbers mentioned throughout this study is available in Additional File 2. Flies were maintained on standard Bloomington food at 18 °C. Crosses were placed in a 25 °C humidified incubator for the entire growth period. All flies were aged for 7 to 10 days prior to sampling. For starvation assays, equal numbers of males and females were transferred into empty vials lined with wetted tissue. Only males were utilized in subsequent experiments.

### Immunohistochemistry and visualization

Anti-Or22a antibodies were produced by Agrisera (Vännas, Sweden). Briefly, the previously reported Or22a peptide sequence PHIKEKPLSERVKSAD [60] was engineered with an additional cysteine at its N terminus, in order to aid subsequent affinity purification. This was injected into a goat host, after which antibodies raised against the Or22a epitope were returned both in whole and purified sera form. These were aliquoted into smaller batches prior to freezing.

Fly heads were arranged in OCT (Sakura® FinTek™, Japan) and immediately placed onto dry ice prior to storage at – 80 °C. Plaques were prepared at least a day ahead of sectioning. Each antennal slice obtained on charged slides (ThermoFisher Scientific, USA) was 10 μm thick. Immunohistochemistry was performed as previously described [61]. The following primary antibodies were used: purified goat anti-Or22a (1:2000), chicken anti-GFP (ab13970, Abcam), mouse anti-21a6 (1:750, Developmental Studies Hybridoma Bank (DSHB)), and rabbit anti-Orco (1:5000, gift from Richard Benton). Secondary antibodies were conjugated to either Alexa Fluor 488 (1:500, Molecular Probes) or Red-X or Far-Red fluorophores (Jackson Technologies, Florida, USA). Images were captured on the SP8 Confocal Microscopy (Leica, Germany) platform at magnifications of 63X and above. We targeted only Or22a OSNs, which occupy a stereotypic region of inner antenna. A standardized setting was locked in for all instances where signal quantification was to be used as the comparative measure. Each maximum intensity projection (MIP) of a Z-stack is comprised of 10 to 12 0.8-μm-thick slices. Images were processed on Photoshop (Adobe Technologies, USA), followed by assisted-quantification on ImageJ (USA). Data obtained and processed from images are found in Additional File 3.

### Quantitative analyses

All images were processed on ImageJ (USA). In colocalization analysis, dual-channel images were split and thresholded appropriately to highlight focal structures. For instance, the threshold for Or22a was adjusted so that only punctate Or22a remained. A macro (developed by D. Richardson, available online on GitHub under 2D_object_colocalization) modified to suit the nature of the sample type was executed to obtain colocalization percentage values. In comparative distribution analysis, the length of an OSN soma was measured from its dendritic to axonal exits and then divided into half crosswise. Each channel was measured directly from raw images and applied to derive dendrite to axon mean gray intensity values. No more than four neurons were isolated and analyzed per antenna in either analysis.

Whole antennal images were utilized in the following analyses. The percentage of soma with puncta was calculated as a function of the number of neurons bearing somatic puncta, at least one of which must be at the dendritic exit, over the total Or22a OSN count. Average puncta frequency was derived from collective somatic and dendritic Or22a puncta count over total Or22a OSN count. For cilia to soma level comparisons, the ciliary and somatic regions of a population of Or22a OSNs were manually isolated and separately analyzed. Background gray leaching was subtracted at an appropriate rolling ball radius. The measured mean gray intensities of each compartment were applied to derive cilia to soma values. All Or22a-positive neurons were included for such analyses without bias, and only one antenna was analyzed per animal. Source data for all analyses can be found in Additional File 3.

### Endo H digestion and Western blot

Antennal lysates were prepared according to a protocol described specifically for the tissue type [62]. Antenna from at least 300 heads were pooled for each sample. Briefly, 20 μg of protein was denatured prior to digestion with Endo H (New England Biolabs, USA) in a sodium citrate buffer (0.05 M, pH 5.5) for 6 h at 37 °C. Once separated and transferred, membranes were blocked prior to overnight probing with primary antibodies at 4 °C. After a series of washing, incubation with secondary antibodies, and treatment with a high-sensitivity detection system (ECL SuperBright, Agrisera, Sweden), the blot was visualized on the ChemiDoc MP Imaging System (Bio-Rad, USA). To note, the blocking and nutation steps for goat-derived antibodies were performed with EveryBlot Blocking Buffer (Bio-Rad, USA). For mouse-derived antibodies, the standard 5% non-fat milk in TBST (0.05% Tween-20) solution was applied. Primary antibodies used were purified goat anti Or22a (1:4,000, Agrisera, Sweden; self-commissioned) and mouse anti α-Tubulin (1:5,000, #12G10, DSHB). HRP-conjugated secondary antibodies used were donkey anti-goat (1:5000, ab6885, Abcam) and donkey anti-mouse (1:5000, ab6820, Abcam). Quantification was performed manually on ImageJ (USA). Source data can be found in Additional File 3.

### Supplementary Information

**Additional file 1.** Supplementary figures S1 to S7. **Fig S1.** Or22a puncta do not overlap with cilia transport pathway markers. **Fig S2.** Or22a puncta are observed at stereotypical sites and do not overlap with ERGIC-53 and Golgi. **Fig S3.** Or22a is not always coupled with Orco in the soma. **Fig S4.** Knockdown of Grasp65 in Or22a OSNs causes a loss in somatic Or22a-puncta but does not affect overall Or22a transport into the cilia. **Fig S5.** Or22a is digestible by Endo H. **Fig S6.** Starvation encourages the Grasp65-linked unconventional transport of Or22a. **Fig S7.** Raw images for Or22a Endo H digestion assays, Figure S5.

**Additional file 2.** Genotype and stock numbers list.

**Additional file 3.** Source data for main and supplementary figures.

## Data Availability

All data generated or analyzed during this study are included in this published article and its supplementary information files. Supplementary figures are in Additional File 1. Genotypes and fly stocks are listed in Additional File 2. Data utilized in analyses can be obtained from Additional file 3. Materials generated by this study such as the goat anti-Or22a antibody can be made available upon request.

## Abbreviations

OSN: Olfactory sensory neuron
OR: Odorant receptor
ERGIC: ER-Golgi intermediate compartment
ER: Endoplasmic reticulum
GFP: Green fluorescent protein
IFT: Intraflagellar transport
BBS: Bardet-Biedl syndrome
UPS: Unconventional protein secretion
CUPS: Compartments of unconventional protein secretion
MVB: Multivesicular body
GRASP: Golgi reassembly and stacking protein
COP: Coat protein complex
Endo H: Endoglycosidase H
