# Supplementary material for: A cilia-bound unconventional secretory pathway for Drosophila odorant receptors
Source: BMC Biol. 2024 Apr 12;22:84. doi: 10.1186/s12915-024-01877-2 (PMC11015608; doi:10.1186/s12915-024-01877-2)
Supplement: Supplementary file 1 — Additional file 1. Supplementary figures S1 to S7. Fig S1. Or22a puncta do not overlap with cilia transport pathway markers. Fig S2. Or22a puncta are observed at stereotypical sites and do not overlap with ERGIC-53 and Golgi. Fig S3. Or22a is not always coupled with Orco in the soma. Fig S4. Knockdown of Grasp65 in Or22a OSNs causes a loss in somatic Or22a-puncta but does not affect overall Or22a transport into the cilia. Fig S5. Or22a is digestible by Endo H. Fig S6. Starvation encourages the Grasp65-linked unconventional transport of Or22a. Fig S7. Raw images for Or22a Endo H digestion assays, Figure S5. [file 12915_2024_1877_MOESM1_ESM.pdf]

Figure S1

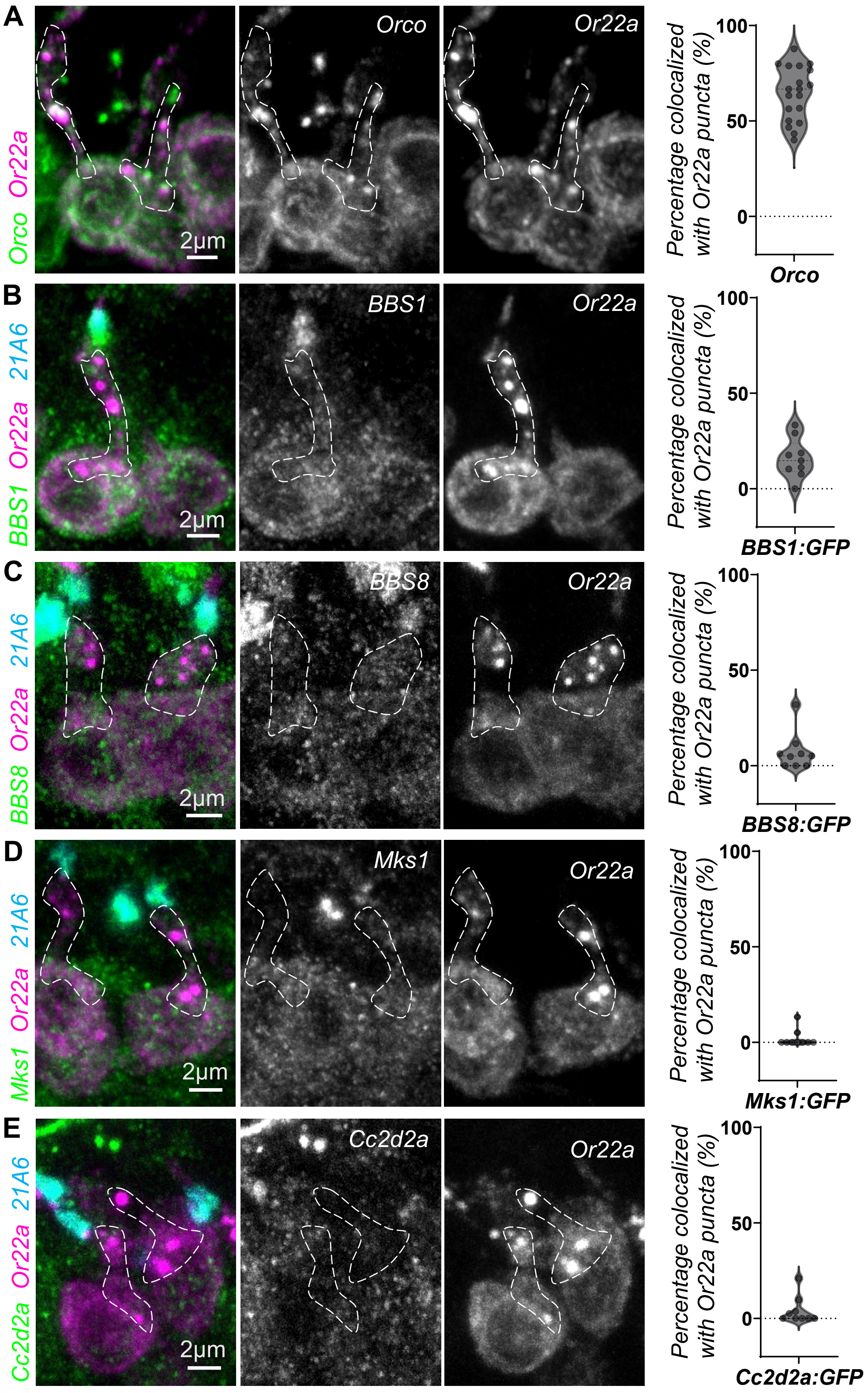
A
Orco
Or22a
Or22a
2µm
Percentage colocalized with Or22a puncta (%)
100
50
0
Orco
B
21A6
Or22a
BBS1
BBS1
Or22a
2µm
Percentage colocalized with Or22a puncta (%)
100
50
0
BBS1:GFP
C
21A6
Or22a
BBS8
BBS8
Or22a
2µm
Percentage colocalized with Or22a puncta (%)
100
50
0
BBS8:GFP
D
21A6
Or22a
Mks1
Mks1
Or22a
2µm
Percentage colocalized with Or22a puncta (%)
100
50
0
Mks1:GFP
E
21A6
Or22a
Cc2d2a
Cc2d2a
Or22a
2µm
Percentage colocalized with Or22a puncta (%)
100
50
0
Cc2d2a:GFP

Figure S1. **Or22a puncta do not overlap with cilia transport pathway markers.**

(A) Or22a colocalizes extensively with Orco (n = 19 puncta).

(B and C) Or22a puncta do not significantly associate with GFP-tagged protein markers of the *Bardet-Biedl Syndrome* BBSome machinery, *BBS1* (B) or *BBS8* (C).

(D and E) Or22a puncta do not significantly associate with GFP-tagged protein markers of cilia transport pathway proteins related to the ciliopathies *Meckel* and Joubert Syndromes i.e. *Mks1* (D) and *Coiled-Coil And C2 Domain Containing 2A, Cc2d2a* (E).

Scale bar in coloured photo represents both preceding grayscale images. 21A6 highlights the cilia base. White dashes outline Or22a puncta, and serve as a visual guide. For each GFP-tagged marker, n = 9 puncta. One-way ANOVA with Orco as the colocalization control returned a $p<0.0001$ for all markers.

Figure S2

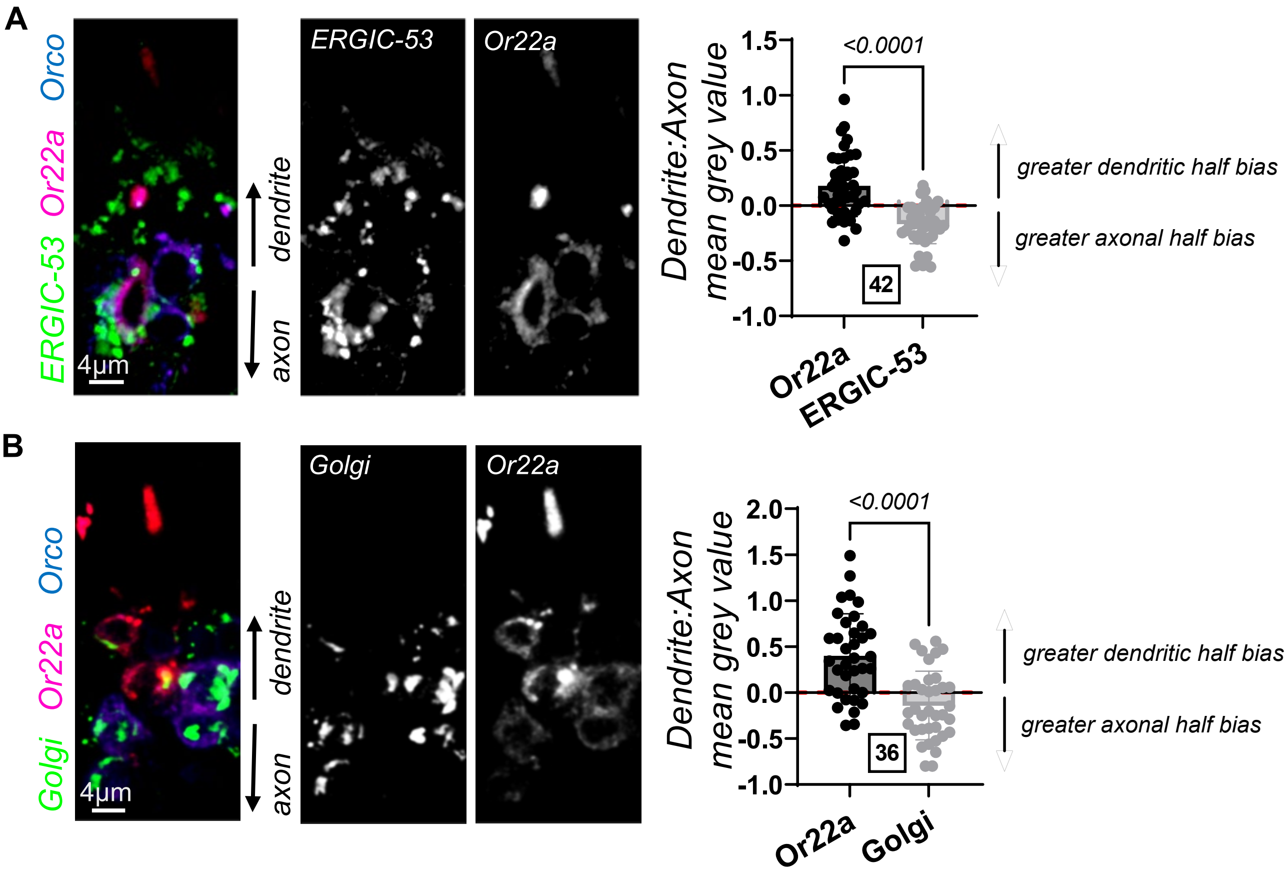

Figure S2. **Or22a puncta are observed at stereotypical sites and do not overlap with ERGIC-53 and Golgi.**

(A and B) Or22a puncta likely originates from the ER, but do not significantly associate with either of downstream conventional protein transport organelles i.e. ERGIC (A) or Golgi (B) structures. Notably, both ERGIC-53, an ERGIC marker, and proteins of the Golgi stack localize with a bias towards the axonal end of the OSN, rather than the dendritic end. The boxed number on the graphs represent total neurons analysed. Error bars represent Mean SEM. Two-tailed Student's T-test was applied for statistical analysis; $p<0.0001$ = ****.

Figure S3

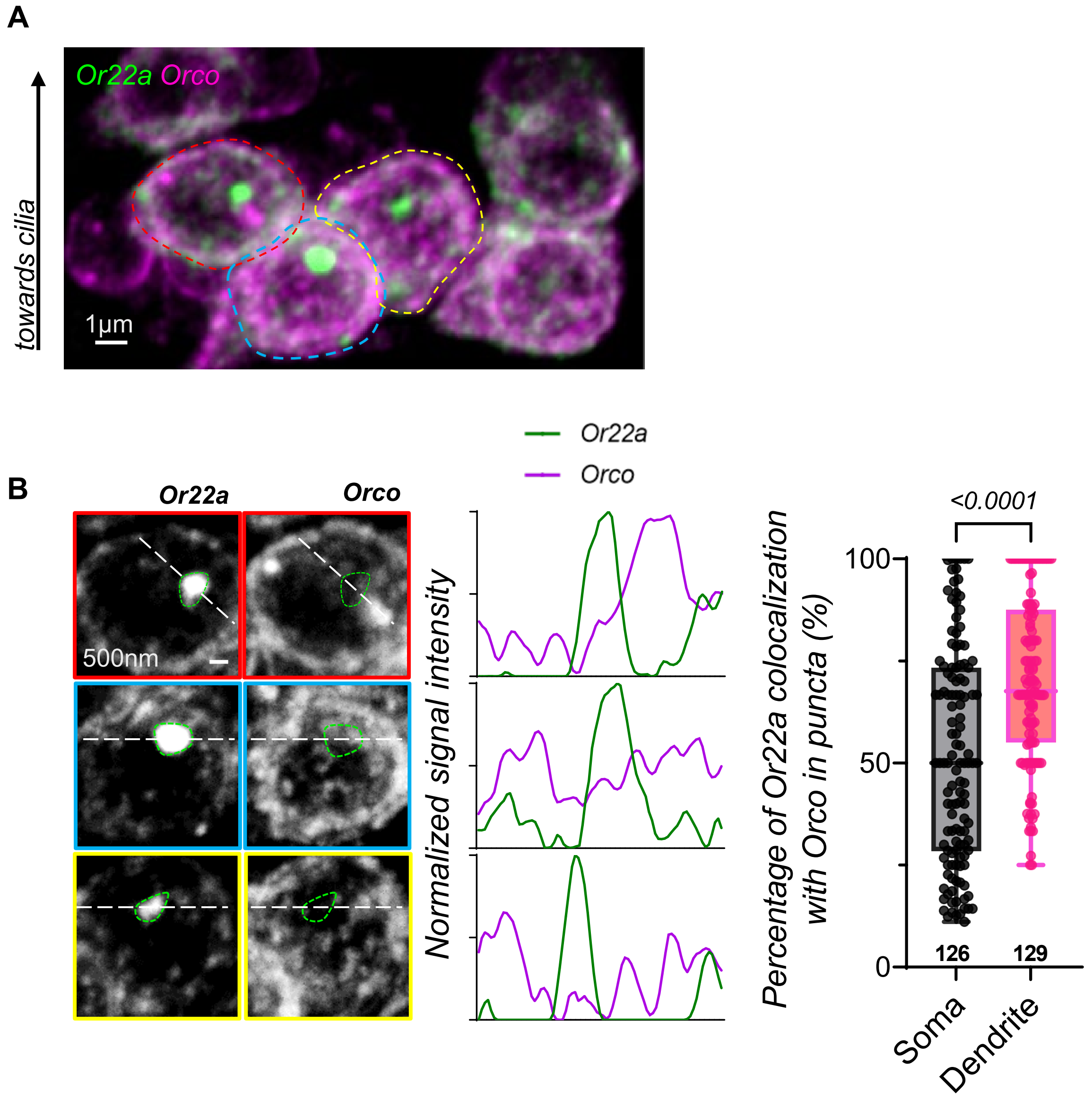

Figure S3. **Or22a is not always coupled with Orco in the soma.**

(A) ORs are assumed to be dependent on Orco for transport. However, Or22a can often be observed as separate entities to Orco in the cell body.

(B) Magnified images correspond by colour to outlined cells. Or22a puncta examined are outlined by green dashes. Histogram profiles are generated from the path of white-dashed lines. The scale bar in the topmost row of applies to succeeding rows. Rightmost graph demonstrates that Or22a colocalizes significantly less perfectly with Orco in the soma compared to the dendrite. Numbers indicate the number of Or22a-positive puncta analysed. Error bars represent Mean SEM. Two-tailed Student's T-test was applied for statistical analysis; $p<0.0001$ = ****

Figure S4

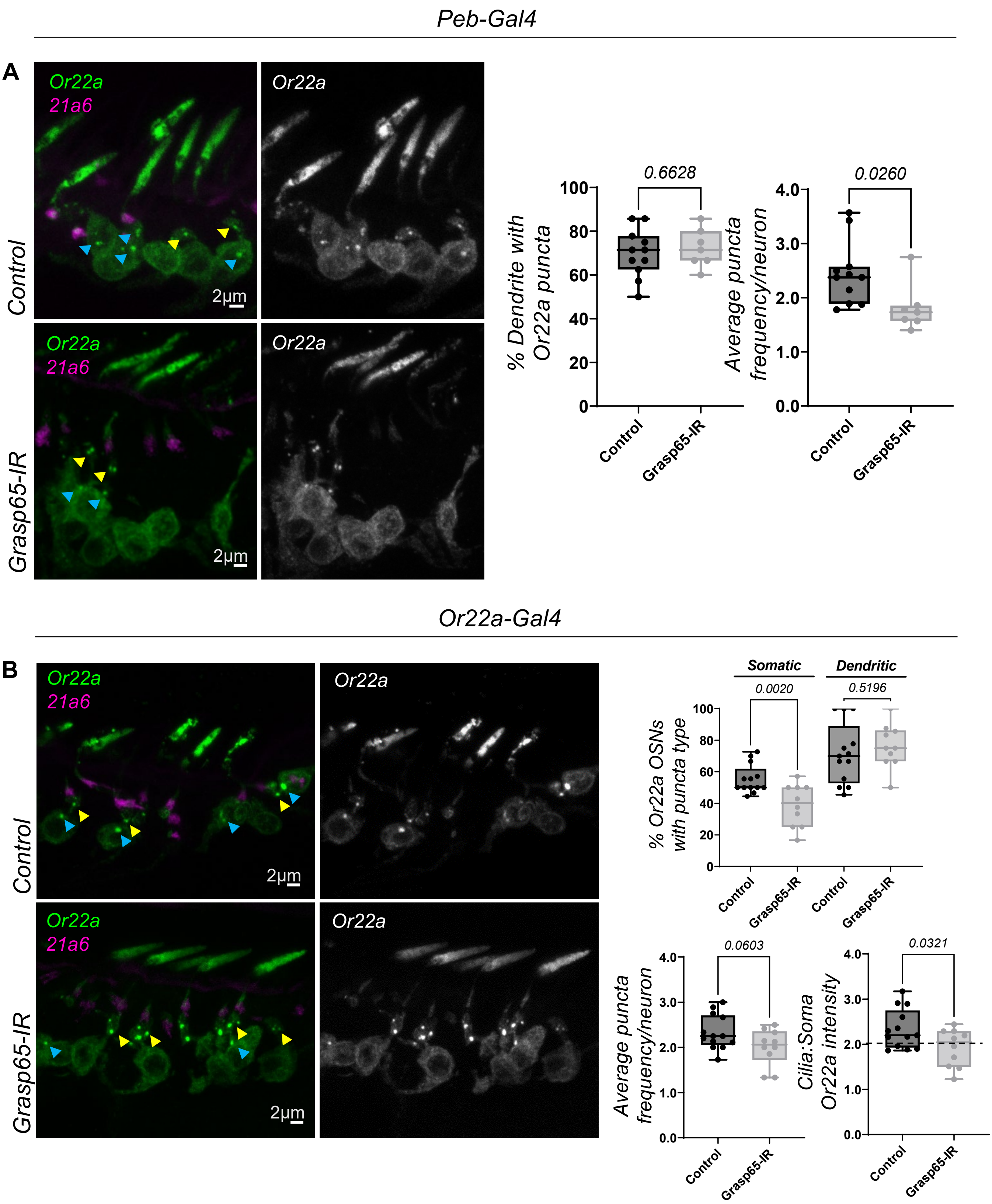

Peb-Gal4
A
Or22a
21a6
Or22a
Control
Grasp65-IR
2μm
% Dendrite with Or22a puncta
0.6628
0.0260
Average puncta frequency/neuron
Control
Grasp65-IR
Or22a-Gal4
B
Somatic
Dendritic
0.0020
0.5196
% Or22a OSNs with puncta type
0.0603
0.0321
Cilia:Soma Or22a intensity

Figure S4. **Knockdown of *Grasp65* in Or22a OSNs causes a loss in somatic Or22a-puncta but does not affect overall Or22a transport into the cilia.**

The loss of *Grasp65* via two OSN-driven Gal4 lines commonly reduces somatic puncta numbers (for *Peb*-Gal4, refer to Figures 4E and 4F) and induces a downward trend in average overall puncta frequency per neuron, but does not reduce ciliary Or22a transport nor dendritic puncta formation.

(A) Representative images of *Peb*-Gal4 driven expression of Control or *Grasp65*-IR in Or22a OSNs. For Control, n =11; for *Grasp65*-IR, n = 7.

(B) Representative images of *Or22a*-Gal4 driven expression of Control or *Grasp65*-IR in Or22a. For Control, n =13; for *Grasp65*-IR, n = 10.

Soma puncta are marked by blue arrowheads, and dendritic puncta by yellow arrowheads. Scale bars are as shown. Error bars represent Mean SEM. Two-tailed Student's T-test was applied for statistical analysis; p>0.05 = no significance, p<0.05 = *, p<0.01 = **

Figure S5

A

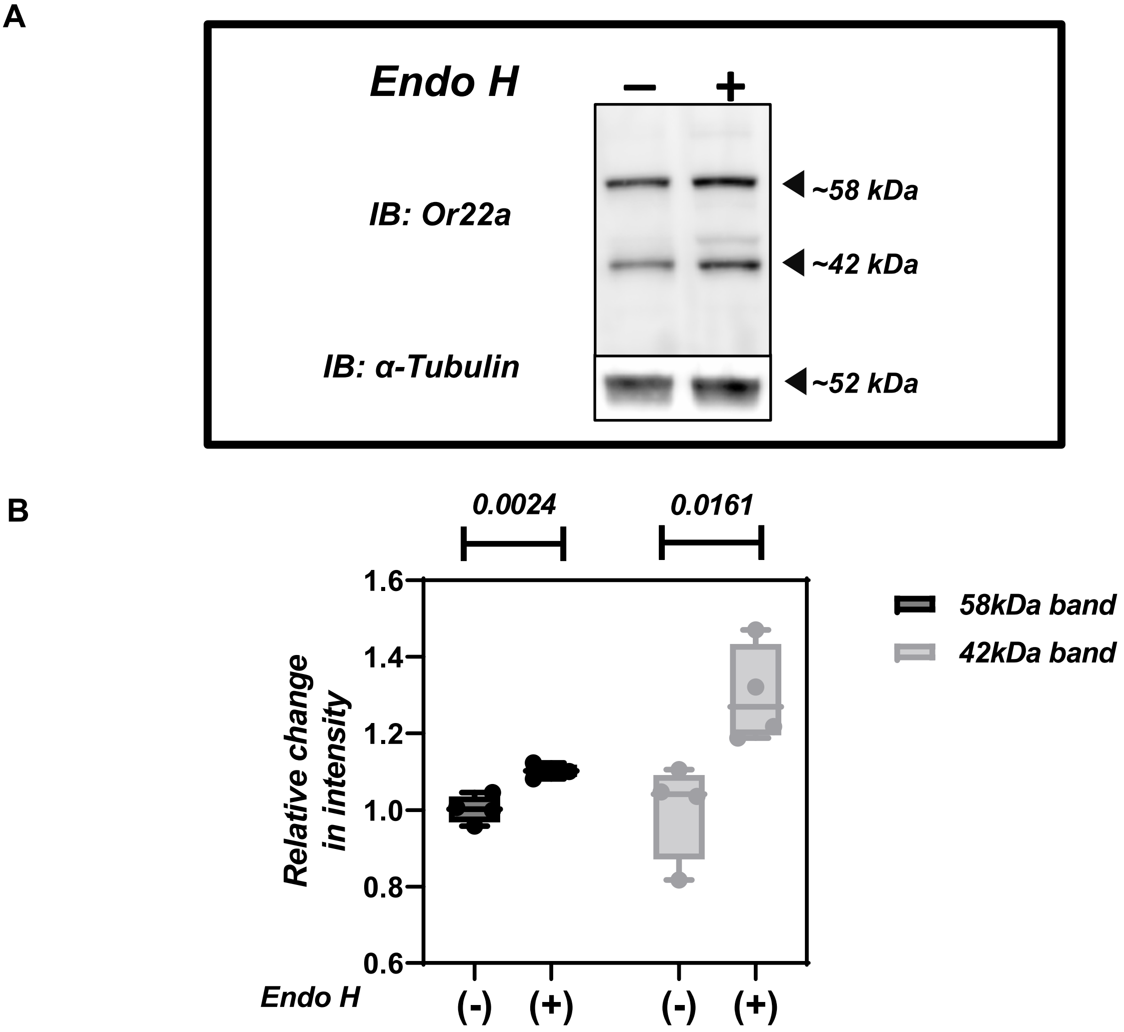

Figure S5. **Or22a is digestible by Endo H.**

(A) Head lysates from wild-type male flies were digested with Endo H ('+' lane) and probed with a purified goat antibody against Or22a. Two distinct bands are highlighted. Digestibility by Endo H is demonstrated by the increased intensities of both bands.

(B) Quantitative analyses of Or22a intensities before and after Endo H digestion (n = 4).

Error bars represent Mean SEM. One sample one-tailed T-tests were applied for statistical analysis; p>0.05 = no significance, p<0. 05 = *, p<0. 001 = ***

Figure S6

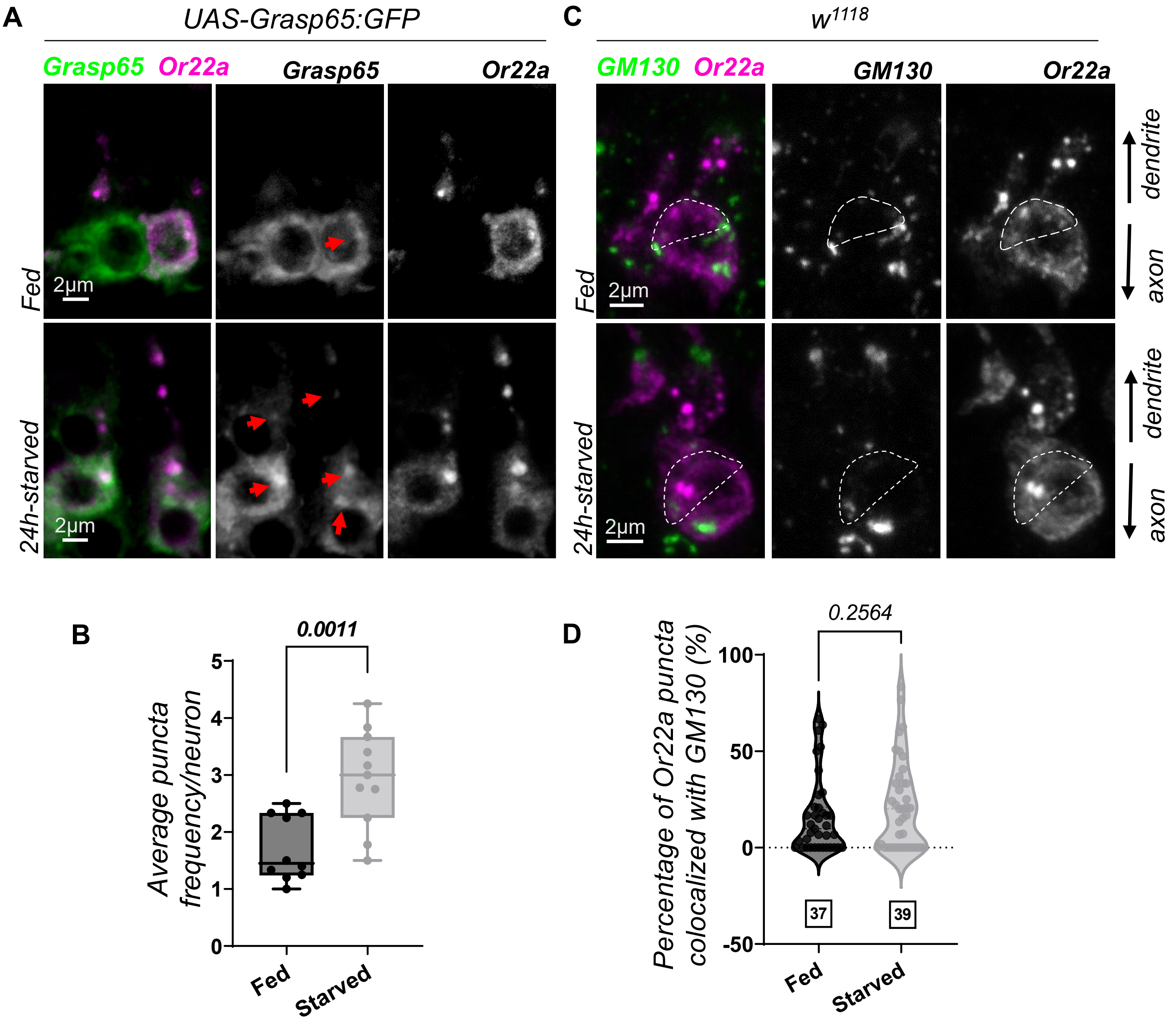

A
UAS-Grasp65:GFP
Grasp65 Or22a
Grasp65
Or22a
Fed
24h-starved
2μm
C
w1118
GM130 Or22a
GM130
Or22a
dendrite
axon
B
0.0011
Average puncta frequency/neuron
Fed
Starved
D
0.2564
Percentage of Or22a puncta colocalized with GM130 (%)
37
39
Fed
Starved

Figure S6. **Starvation encourages the Grasp65-linked unconventional transport of Or22a**.

(A) A 24-hour period of food deprivation increased the number of Grasp65-overlapping Or22a puncta.

(B) Quantitative analysis of puncta frequency. For fed flies, n = 10; for starved flies, n = 11.

(C) Increased Or22a puncta numbers from starvation is not due to an increased association to the *cis*-Golgi, marked by GM130. The dendritic-half of the soma is outlined in white.

(D) Quantitative analysis of colocalization between Or22a puncta and GM130. The boxed number indicate the number of neurons sampled.

Error bars represent Mean SEM. Two-tailed Student's T-test was applied for statistical analysis; $p>0.05$ = no significance, $p<0.01$ = **

Figure S7

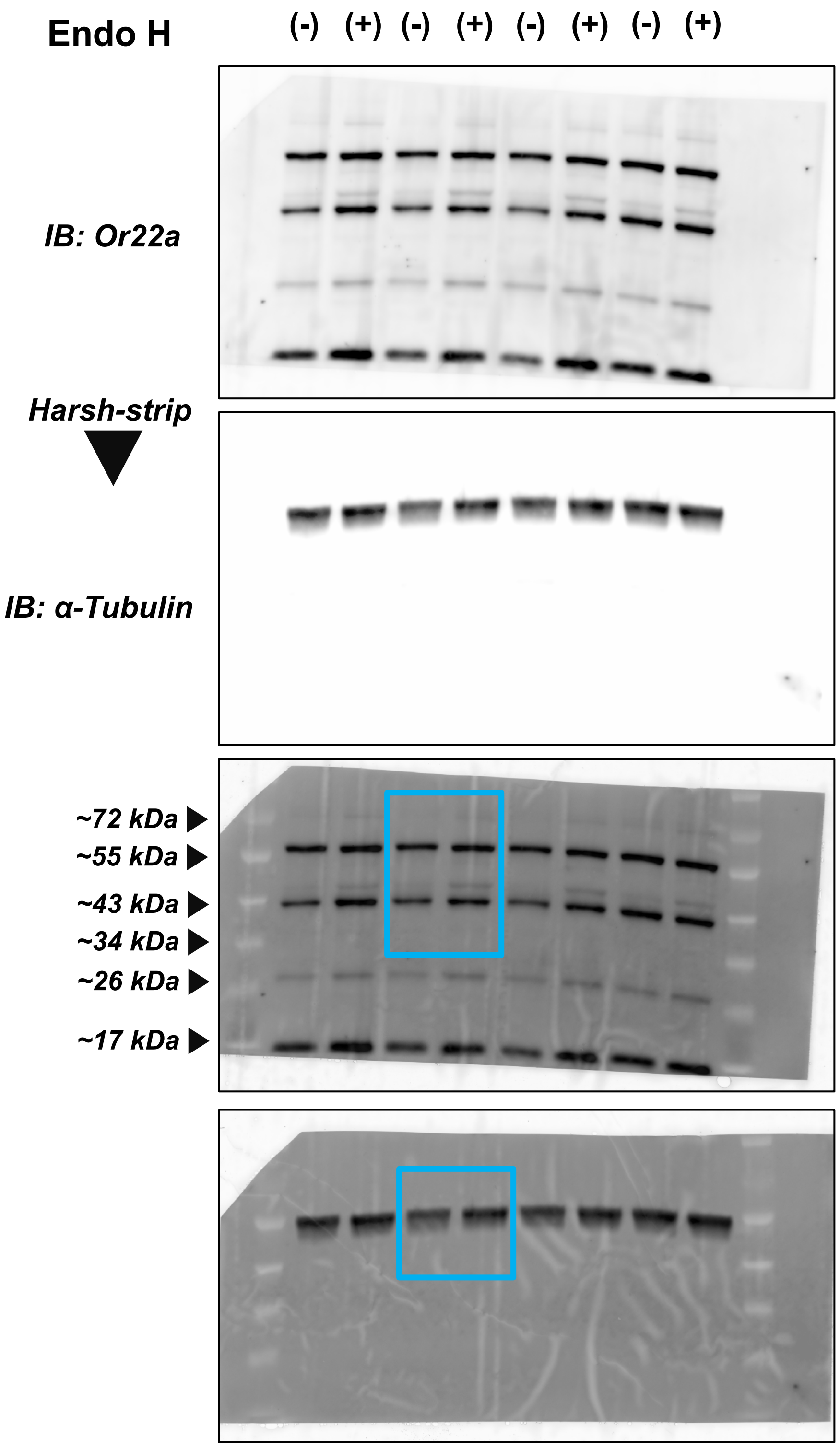

Figure S7. **Raw images for Or22a Endo H digestion assays, Figure S5**

Antennal extracts run on 4 – 20% precast gels. The bottom two images are superimposed with whole light images to indicate size and show that both Or22a and the control were probed on a singular membrane. All antennal lysates were obtained from w^{1118} flies. A lane marked (-) indicates an undigested lysate, with the (+) lane to its right indicating an Endo H digested lysate. The bioreplicate chosen for Figure S5 is boxed blue.
