# Supplementary material for: A cilia-bound unconventional secretory pathway for Drosophila odorant receptors
Source: BMC Biol. 2024 Apr 12;22:84. doi: 10.1186/s12915-024-01877-2 (PMC11015608; doi:10.1186/s12915-024-01877-2)
Supplement: Supplementary file 2 — Additional file 2. Genotype and stock numbers list. [file 12915_2024_1877_MOESM2_ESM.pdf]

## Genotype and Source list

**Figure 1. (A) Wild-type :** *$w^{1118}$ / Y ; + / + ; + / +.* **(B) NompB:GFP :** *$w^{1118}$ ; NompB:GFP / CyO ; + / +.* **(C) ERGIC-53:GFP :** *$w^{1118}$ ; + / + ; dERGIC53:GFP / dERGIC53:GFP.* **(D) Golgi:GFP :** *$w^{1118}$ ; + / + ; sqh-EYFP:Golgi / sqh-EYFP:Golgi.*

**Figure 2. (B) Control :** *Peb-Gal4, UAS-Dcr2 / Y ; + / + ; + / +.* ***Sec16-IR*** **:** *Peb-Gal4, UAS-Dcr2 / Y ; UAS-Sec16-IR / + ; + / +.* ***Sec22-IR*** **:** *Peb-Gal4, UAS-Dcr2 / Y ; + / + ; UAS-Sec22-IR / +.* ***Sec31-IR*** **:** *Peb-Gal4, UAS-Dcr2 / Y ; + / + ; UAS-Sec31-IR / +.* ***Syx5-IR*** **:** *Peb-Gal4, UAS-Dcr2 / Y ; + / + ; UAS-Syx5-IR / +.* ***Sec23-IR*** **:** *Peb-Gal4, UAS-Dcr2 / Y ; + / + ; UAS-Sec23-IR / +.*

**Figure 3. (A and C) Control :** *Peb-Gal4, UAS-Dcr2 / Y ; + / + ; + / +.* **(B and D)** ***ERGIC-53-IR*** **:** *Peb-Gal4, UAS-Dcr2 / Y ; UAS-ERGIC53-IR / + ; + / +.*

**Figure 4. (A and B)** ***Grasp65:GFP*** **:** *Peb-Gal4, UAS-Dcr2 / Y ; UAS-Grasp65:GFP / + ; + / +.* **(C) Control :** *Peb-Gal4, UAS-Dcr2 / Y ; + / + ; + / +.* **(D)** ***Grasp65-IR*** **:** *Peb-Gal4, UAS-Dcr2 / Y ; + / + ; UAS-Grasp65-IR / +.*

**Figure 5. (A and D) Wild-type :** *$w^{1118}$ / Y ; + / + ; + / +.*

**Figure 6. (C) Wild-type :** *$w^{1118}$ / Y ; + / + ; + / +.* ***Grasp65$^{[102]}$*** **:** *w[*]; wg[Sp-1]/CyO; Grasp65$^{[102]}$*. ***GM130$^{[\Delta 23]}$*** **:** *w[*]; GM130$^{[\Delta 23]}$; TM2/TM6B, Tb[1].*

**Figure S1. (A) Wild-type :** *$w^{1118}$ / Y ; + / + ; + / +.* **(B) BBS1:GFP :** *$w^{1118}$ ; BBS1:GFP / BBS1:GFP ; + / +.* **(C) BBS8:GFP :** *$w^{1118}$ ; + / + ; BBS8:GFP / BBS8:GFP.* **(D) Mks1:GFP :** *w* ; + / + ; Mks1:GFP / Mks1:GFP.* **(E) Cc2d2a:GFP :** *$w^{*}$ ; + / + ; Cc2d2a:GFP / Cc2d2a:GFP.*

**Figure S2.** +. **(A) ERGIC-53:GFP :** *$w^{1118}$ ; + / + ; dERGIC53:GFP / dERGIC53:GFP.* **(B) Golgi:GFP :** *$w^{1118}$ ; + / + ; sqh-EYFP:Golgi / sqh-EYFP:Golgi.*

**Figure S3. (A and B) Wild-type :** *$w^{1118}$ / Y ; + / + ; + / +.*

**Figure S4. (A) Control :** *Peb-Gal4, UAS-Dcr2 / Y ; + / + ; + / +.* ***Grasp65-IR*** **:** *Peb-Gal4, UAS-Dcr2 / Y ; + / + ; UAS-Grasp65-IR / +.* **(B) Control :** *$w^{1118}$ / Y ; + / + ; Or22a-Gal4 / +.* ***Grasp65-IR*** **:** *+ / Y ; + / + ; Or22a-Gal4 / UAS-Grasp65-IR.*

**Figure S5. (A) Wild-type :** *$w^{1118}$ / Y ; + / + ; + / +.*

**Figure S6. (A)** ***Grasp65:GFP*** **:** *Peb-Gal4, UAS-Dcr2 / Y ; UAS-Grasp65:GFP / + ; + / +.* **(C) Wild-type :** *$w^{1118}$ / Y ; + / + ; + / +.*

**Figure S7. For all lanes:** *$w^{1118}$ / Y ; + / + ; + / +.*

| Ref. Name | Source, Line# | Figure(s) |
|---|---|---|
| ***NompB:GFP*** | Donated | ***1B*** |
| ***ERGIC-53:GFP*** | VDRC, #318063 | ***1C, S2A*** |
| ***Golgi:GFP*** | BDSC, #7193 | ***1D, S2B*** |
| ***Sec16-IR*** | BDSC, #53917 | ***2B*** |
| ***Sec22-IR*** | BDSC, #34893 | ***2B*** |
| ***Sec31-IR*** | BDSC, #32878 | ***2B*** |
| ***Syx5-IR*** | BDSC, #29397 | ***2B*** |
| ***Sec23-IR*** | BDSC, #32365 | ***2B*** |
| ***ERGIC-53-IR*** | BDSC, #55657 | ***3B, 3D*** |
| ***Grasp65:GFP*** | BDSC, #8507 | ***4A, 4B, S6A*** |
| ***Grasp65-IR*** | BDSC, #34082 | ***4D, S4A, S4B*** |
| ***Or22a-Gal4*** | BDSC, #9951 | ***S4B*** |
| ***Grasp65***$^{[102]}$ | BDSC, # 65257 | ***6C*** |
| ***GM130***$^{[\Delta 23]}$ | BDSC, # 65255 | ***6C*** |
| ***BBS1:GFP*** | VDRC, #318684 | ***S1B*** |
| ***BBS8:GFP*** | VDRC, #318636 | ***S1C*** |
| ***Mks1:GFP*** | VDRC, #318529 | ***S1D*** |
| ***Cc2d2a:GFP*** | Donated | ***S1E*** |
